# Epidemiologic Correlates of Mortality among Symptomatic Visceral Leishmaniasis Cases: Findings from Situation Assessment in High Endemic Foci in India

**DOI:** 10.1371/journal.pntd.0005150

**Published:** 2016-11-21

**Authors:** Aritra Das, Morchan Karthick, Shweta Dwivedi, Indranath Banerjee, Tanmay Mahapatra, Sridhar Srikantiah, Indrajit Chaudhuri

**Affiliations:** CARE India Solutions for Sustainable Development. Patna, Bihar, India; University of Minnesota, UNITED STATES

## Abstract

**Background:**

Visceral leishmaniasis (VL) is highly prevalent in the Indian state of Bihar and, without proper diagnosis and treatment, is associated with high fatality. However, lack of efficient reporting mechanism had been an impediment in estimating the burden of mortality and its antecedents among symptomatic VL cases. The objectives of the current study were to generate a reliable estimate of symptomatic VL caseload and mortality in Bihar, as well as to identify the epidemiologic and health infrastructure-related predictors of VL mortality.

**Methodology and Principal Findings:**

Using an elaborate index case tracing method, we attempted to locate all symptomatic VL patients in eight districts of Bihar. Interviews and medical-record-reviews were conducted with cases (or next-of-kin for the dead) meeting the eligibility criteria. The information collected during the interviews included socio-demographic characteristics, onset of disease symptoms, place of diagnosis, pre- and post-diagnosis treatment history, type and duration of drugs received. In total, we analyzed data on 4925 VL patients—59% were male and 68% were less than 30 years old. There were 158 (3.2%) deaths and the incidence rate of mortality was 3.2/100 person-years. In the adjusted Cox-proportional-hazards analysis, treatment at public facility [Adjusted Hazard Ratio (AHR) = 0.61; 95% CI = 0.43–0.86], shorter (≤30 days) diagnostic delay [AHR = 0.62, 95% CI = 0.43–0.92], and treatment completion [AHR = 0.03, 95% CI = 0.02–0.05] emerged as significant negative predictors of mortality.

**Conclusion:**

Mortality reduction efforts in Bihar should focus on improving access to early diagnosis, quality treatment and treatment-adherence measures, with special emphasis on marginalized communities.

## Author Summary

More than 70% cases of visceral leishmaniasis (VL), a parasitic disease transmitted by sand flies, in India are reported from Bihar, a resource-poor state. In absence of early diagnosis and treatment, the fatality is very high among symptomatic VL cases. However, community-based data on VL mortality is limited in Bihar. Also, little information on the factors associated with mortality among symptomatic VL patients is available from this Indian state. Evidences regarding mortality parameters can prove immensely helpful in designing specific interventions targeted at reduction of such mortality. In the current study, we created a registry of symptomatic VL cases in eight districts of Bihar to estimate the burden of VL mortality and the socio-demographic factors associated with it. The mortality rate among the symptomatic VL patients was found to be fairly high. Shorter interval between onset of symptoms and diagnosis, and completing course of treatment were associated with increased survival. We recommend improving access to early diagnosis and treatment services, especially among the marginalized communities, as a key measure to reduce mortality among symptomatic VL patients in Bihar.

## Introduction

Globally, an estimated 500,000 new cases of visceral leishmaniasis (VL) or kala-azar, a ‘Neglected Tropical Disease’, occur annually [1]. More than 90% of the global burden of visceral leishmaniasis (VL) is contributed by six countries: Bangladesh, Brazil, Ethiopia, India, South Sudan and Sudan [2]. The parasite *Leishmania donovani* is the main causative agent of VL in India, Nepal, and Bangladesh, where it is transmitted by the sand fly vector *Phlebotomus argentipes* [3]. In Indian subcontinent, as per the prevailing knowledge of transmission dynamics, humans are considered to be the sole carrier of the disease (anthroponotic) and transmission pattern is believed to be peridomestic i.e. transmitted by the bite of female sand flies in and around the home [4, 5].

Based on recent estimates, in India, 70%–90% VL cases occur in a single state–Bihar [6, 7]. The disease was considered to be at the brink of elimination in Bihar during 1960s following widespread DDT use under National Malaria Eradication Program [8, 9]. However, in absence of a dedicated VL elimination strategy and surveillance system, the disease saw a resurgence a decade later [9, 10]. Since then VL has been widely acknowledged as a major public health concern in India. Still a significant proportion of VL caseload in Bihar was assumed to remain outside the public health detection system until recent past [11, 12]. Absence of a robust surveillance system as well as simple and inexpensive diagnostic tests were identified as potential reasons for the poor tracking of VL cases in Indian subcontinent [11, 13].

In order for an effective kala-azar elimination program in Bihar, identification of high endemic foci is essential. Health Management Information System (HMIS), the current source of population level information on VL, has been reported to suffer from various methodological and operational problems including underreporting and ill-defined population frame [11, 12]. For precise identification of blocks having VL incidence above the elimination level (i.e. annual incidence of 1/10000 population or more), a situation assessment project was undertaken in January’2013 [7]. It was also expected that this assessment would help in evaluating the effectiveness of various elimination strategies (e.g. treatment by amphotericin-B or miltefosine) and inform the kala-azar elimination program in India on the incidence of VL associated mortality and its epidemiologic correlates.

In absence of early diagnosis and treatment, fatality was detected to be very high among symptomatic VL cases [14, 15]. Even among treatment-recipients, the measured probability of dying within two years of the onset of symptoms were quite high [7, 16]. Further, among many diagnosed patients, outcome remained unknown due to lack of systematic follow up and cause of death remained obscured among many suspected cases in the community. Thus, alike incidence, it can be hypothesized that mortality due to VL also remain grossly under-reported. To the best of our knowledge, no large community-based study did yet report on either the incidence of mortality or its antecedents among symptomatic VL cases in India. The current exercise, therefore, was undertaken to address this knowledge gap. Our objectives were to provide a reliable estimate of symptomatic VL case load and mortality in selected districts of Bihar, and to identify the epidemiologic and treatment-related predictors of VL mortality.

## Methods

### Study population and VL case tracking

In 2013, CARE India undertook a *VL situation assessment* project in eight (out of 38) districts (representing both high and low endemic foci) of Bihar. The principal objective of this project was to inform the kala-azar elimination program operations in this resource-poor state. An important component of this undertaking was to estimate the burden of symptomatic VL cases in the selected districts–through detection of such cases and by tracking them to their households. The reference period (period during which VL diagnosis took place) for the assessment was between January 2012 and June 2013. The following combination of methods was followed to meet this objective:

1. **Index case tracing and snowballing:** A roster of possible VL patients, diagnosed at state-run health facilities (block and district hospitals) during the reference period, was created. Additionally, information on potential VL cases from other facilities in the study area, known to specialize in kala-azar care, were added to the above line list. The compiled list was then scrutinized for removal of duplications. An attempt was made to trace each patient in that list using the available address.
  For cases that were successfully traced, the patient or his/her family members were interviewed to determine, inter alia, if they possessed documents related to their diagnosis and treatment for VL, if there were other known cases in the family or neighborhood, or if the family members were aware of any other patients of kala-azar elsewhere. Contact information of such suspected patients was collected. If a case from the line list could not be traced to the designated address, his/her information was shared with the study staff working in other blocks/districts of Bihar. If, after all these efforts, a patient could not be traced, then it was assumed that the case did not belong to the study districts.
  As part of the case finding effort, key informants from the villages mentioned in the address of potential cases were interviewed to determine if they were aware of any other VL patients or cases of any prolonged fever during the reference period. The key informants included community health workers [such as ASHA (Accredited Social Health Activist), AWW (Anganwadi Worker)] and school teachers. Contact information of any such suspected cases was also collected.
2. **Scanning all private providers in the study districts:** All private laboratories and pharmacists in all villages/towns of each of the study districts were mapped and interviewed to find out if they had diagnosed, treated or dispensed medications to any VL patient during the reference period. Similarly, all known private clinics run by qualified doctors and all unqualified practitioners who could be mapped, were approached to find out if they had seen any VL cases (confirmed or with VL like symptoms) during the reference period. All available details of such suspected cases were recorded.

### VL case definition

The following eligibility criteria were used to determine if a potential subject, identified through the above methodology, could be considered a case of VL:

1. Inclusion in the line list of VL patients of a public facility (within the reference period) and traced to listed place of residence, irrespective of possession of documents related to diagnosis or treatment.
2. Possession of document(s), from private or public facility, of either a serologic rapid diagnostic test (RDT) positive for VL or a report of splenic or bone marrow biopsy positive for the parasite. Additionally, it was checked if the date of diagnosis fell within the study reference period.
3. Possession of document(s), including prescriptions/pharmacy slips/drug packaging, indicating treatment with miltefosine, sodium stibogluconate (SSG) or amphotericin B and start of treatment falling in the reference period.

### Participant interview

Study investigators conducted face-to-face interviews with every VL cases who were identified by the above methodology and met the eligibility criteria. In case a patient died in the interim, interview was conducted with the next-of-kin. The information collected during the interviews included socio-demographic characteristics, onset of symptoms, place of diagnosis, pre- and post-diagnosis treatment history, type and duration of drugs received etc. If available, information on death was obtained from medical documents, else we relied on the information provided by interviewee. The date of onset of symptoms was based on recall. Local calendars and list of local festivals were used by the study investigators to facilitate participants’ recall and to estimate the approximate dates. Respondents who did not possess any treatment-related documents were asked about the treatment regimen. At the time of conduction of this study, three anti-leishmanial drugs were available in the study area–SSG, amphotericin B, and miltefosine. If intake of oral drugs was reported, then it was assumed that the patient had received miltefosine. In case of treatment with injectables, depending on the reported regimen, either SSG or amphotericin B was decided. The breakdown of frequencies regarding source of drug information is presented in supporting information S1 Table. Informed verbal consent was obtained from all adult respondents before collecting the information. If the respondent was a minor, then informed consent was obtained from the respective parent/caregiver.

### Statistical analysis

CS Pro 5.0 was used for data entry, assessments of logical consistencies and detection of duplicate entries. Descriptive analyses were carried out to determine the distribution of socio-demographic and disease-related characteristics of the study population. Bivariate statistical tests (Pearson chi-square) were performed to check for differences in the characteristics of dead and surviving VL patients.

Kaplan-Meier survival curves were constructed and long-rank tests were performed to compare the survival probabilities (from the day of VL diagnosis) across the categories of various demographic and treatment related factors. For identification of the factors associated with deaths among VL cases, unadjusted and adjusted hazard ratios (HR) were obtained by employing Cox proportional-hazards (CPH) models. To account for subject-wise variabilities in the interval between the dates of VL diagnosis and interview, we used a censoring method similar to that employed for cohort studies (deaths were considered as failure events and surviving cases were right censored on the date of interview). In the unadjusted analyses, we assessed the association between death and type of treatment facility (public or private) place of diagnosis (public or private), interval between onset of symptoms and VL diagnosis (≤30 days and >30 days), type of drug used for treatment (amphotericin-B, miltefosine or SSG), and completion of treatment course. The hazard ratios for each of the five predictors, adjusted for patient’s age, gender, caste and socio-economic status, were assessed using five separate CPH models. According to caste, patients were categorized into–marginalized (scheduled castes and scheduled tribes), semi-marginalized (other backward castes) and general/other caste. Regarding socio-economic status, we summarized the information of three variables–type of house, ownership of cattle and belonging to ‘mahadalit’ caste (poorest of poor)–using principal component analysis. We used the derived principal components as surrogate measure for socio-economic status. The proportional hazards assumption was tested using Schoenfeld residuals method. Time dependent covariates were created from the independent variables used in CPH models. For each CPH model, it was assessed if these time dependent covariates had non-zero slopes in a generalized linear regression of the scaled Schoenfeld residuals on functions of time. SAS version 9.4 was used for statistical analyses.

### Ethical approval

The current study was approved by the Institutional Committee for Ethics and Review of Health Management Research Office of Indian Institute of Health Management Research (IIHMR), Jaipur, India (www.iihmr.org). Informed consent (including signature or left thumb impression of the respondent) was obtained from each agreeing participant before interview and measurements, after explaining the details of the study in a language that they could understand.

## Results

Through the index case tracing method, 5770 listed VL cases were identified from the public facilities. Among them, 4962 cases (or their next-of-kin for those who were dead) were successfully traced to their residence. Additionally, records of 1119 cases meeting the study eligibility criteria were obtained from private facilities and through snowballing technique. Following removal of duplicate listings, total 5432 eligible cases were thus recruited. Further, for the current analysis, we did not include i) cases with post-kala-azar dermal leishmaniasis, ii) cases with ambiguous mortality data, and iii) cases for which reported onset of symptoms occurred more than two years before date of diagnosis (because of reliability of recall). Thus, another 507 cases were excluded from the final dataset. Therefore, information on 4925 VL cases who were diagnosed between January 2012 and June 2013 was used for the current exercise. The distribution of cases identified through different sources and at various stages of the study is presented in supporting information S2 Table.

The mean age of the VL patients was 24.9 years (SD ±17.6 years) and about 59% of them were males. More than 80% belonged to either marginalized or semi-marginalized castes. About two-thirds (67%) of the eligible respondents lived in mud/thatched (Kaccha) houses. Majority of the cases not only got tested for VL at a public laboratory (63%) but also received treatment exclusively from public facilities (78%). About 5% patients did not complete their course of treatment, with proportion of treatment defaulter being higher among those prescribed SSG (8%) compared to miltefosine (4%) or amphotericin-B (4%). About 3% VL patients died during the interval between diagnosis and case tracing. The incidence rate of death among symptomatic VL patients was 3.2/100 person-years. The socio-demographic and disease-related characteristics of VL patients are depicted in Table 1.

**Table 1:** Comparison of socio-demographic and disease-related characteristics between the dead and surviving (till date of interview) VL cases in 8 districts of Bihar. 2012–13 (N = 4925)

Characteristics of the respondents		Frequency (%)			*P*-value*
		Alive (n = 4767)	Died (n = 158)	Total	
Age of the patient					
	< = 5 years	375 (7.87)	16 (10.13)	391 (7.94)	Reference
	6–15 years	1652 (34.65)	17 (10.76)	1669 (33.89)	**<0.0001**
	16–30 years	1248 (26.18)	25 (15.82)	1273 (25.85)	**0.0202**
	31–45 years	860 (18.04)	36 (22.78)	896 (18.19)	0.9504
	46–60 years	487 (10.22)	44 (27.85)	531 (10.78)	**0.0124**
	> 60 years	145 (3.04)	20 (12.66)	165 (3.35)	**0.0008**
Gender					
	Male	2814 (59.03)	103 (65.19)	2917 (59.23)	Reference
	Female	1953 (40.97)	55 (34.81)	2008 (40.77)	0.1212
Caste*					
	General	926 (19.43)	25 (15.82)	951 (19.31)	Reference
	Semi-marginalized	2086 (43.76)	59 (37.34)	2145 (43.55)	0.8473
	Marginalized	1755 (36.82)	74 (46.84)	1829 (37.14)	0.0576
Type of house**					
	Kaccha	3202 (67.17)	101 (63.92)	3303 (67.07)	Reference
	Semi-pucca	1096 (22.99)	40 (25.32)	1136 (23.07)	0.4428
	Pucca	469 (9.84)	17 (10.76)	486 (9.87)	0.6023
Family owns cattle					
	Yes	2940 (61.67)	80 (50.96)	3020 (61.33)	Reference
	No	1827 (38.33)	77 (49.04)	1904 (38.67)	**0.0067**
Cattle-shed within the premise					
	Yes	1334 (27.98)	40 (25.32)	1374 (27.9)	Reference
	No	3433 (72.02)	118 (74.68)	3551 (72.1)	0.4621
Interval between start of VL symptoms and diagnosis					
	≤ 30 days	2038 (42.75)	53 (33.54)	2091 (42.46)	Reference
	> 30 days	2729 (57.25)	105 (66.46)	2834 (57.54)	**0.0212**
Place of VL testing					
	Public facility	2984 (63.07)	101 (64.33)	3085 (63.11)	Reference
	Private facility/laboratory	1747 (36.93)	56 (35.67)	1803 (36.89)	0.748
Place of treatment for VL					
	Treated only at public facilities	3751 (78.69)	104 (65.82)	3855 (78.27)	Reference
	Treated partly or completely at private facilities	1016 (21.31)	54 (34.18)	1070 (21.73)	**0.0001**
Drug received					
	Amphotericin-B	373 (8.13)	15 (11.03)	388 (8.22)	0.2152
	Miltefosine	3869 (84.37)	110 (80.88)	3979 (84.27)	Reference
	Sodium stibogluconate	344 (7.5)	11 (8.09)	355 (7.52)	0.7145
Completion of treatment course					
	Yes	4618 (96.87)	67 (42.41)	4685 (95.13)	Reference
	No	149 (3.13)	91 (57.59)	240 (4.87)	**<0.0001**

The numbers may not add up to total because of missing values.

*P-value for comparison between the characteristics of the dead and surviving patients (*X*^*2*^ test)

*P*-values in bold indicate statistical significance (<0.05)

**Fig 1:**
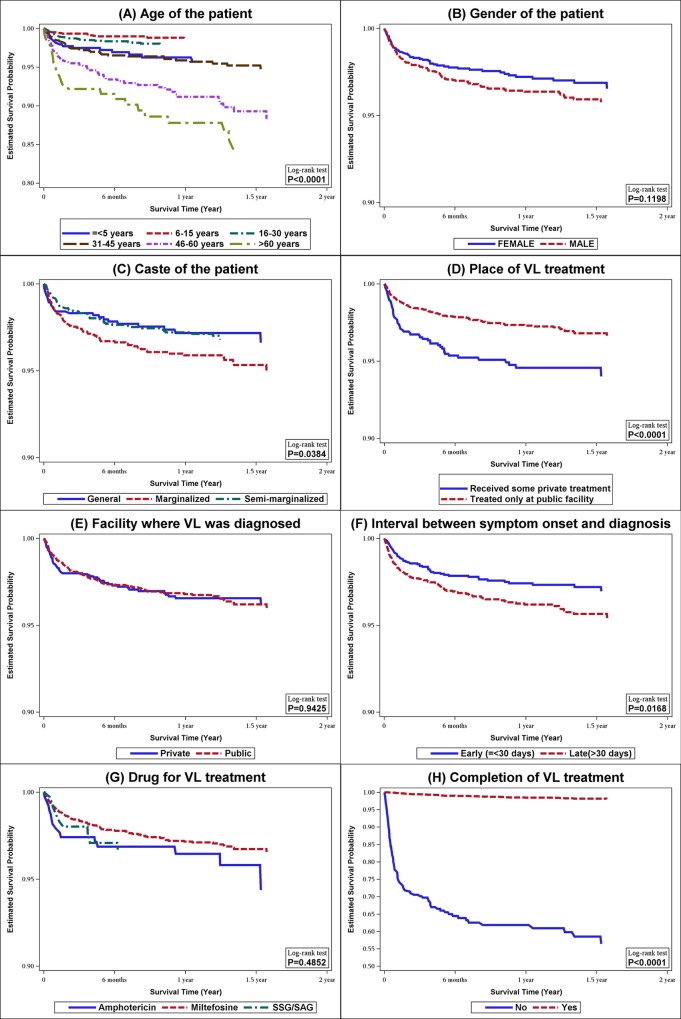
Estimates of the cumulative survival probabilities (Kaplan-Meier estimates) of VL patients, from the time of diagnosis, stratified by the categories of: (A) age of the patient; (B) gender of the patient; (C) caste of the patient; (D) place from which VL treatment was received; (E) facility where VL was diagnosed; (F) interval between symptom onset and diagnosis; (G) type of drug used for VL treatment; and (H) completion of the course of VL treatment. Bihar, 2012–13. (N = 4925).

Bivariate comparison of the characteristics between the dead and the alive (till the date of interview) VL patients revealed that there were significant differences in age (higher proportion of older patients among the dead) and caste (higher proportion of marginalized caste among the dead) distribution between the dead and the alive (Table 1). Moreover, patients who completed the course of their therapy, received earlier diagnosis of VL and those who received treatment from the public facilities were less likely to die. Regarding Kaplan-Meier estimates of the probability of survival in the period following diagnosis (Fig 1), there were statistically significant differences (log rank tests) in cumulative survival probabilities across the categories of age, caste, treatment facility, diagnosis interval and treatment completion.

Table 2 depicts the hazard ratios (and 95% confidence intervals) of death among VL patients associated with various predictors. Those who received treatment exclusively from public facilities were found to have lower risks of death compared to those treated (completely or partially) at private facilities [Unadjusted hazard ratio (HR) = 0.52; 95% confidence interval (CI) = 0.37, 0.72; Adjusted: HR = 0.61; 95% CI = 0.43, 0.86]. Getting an early diagnosis (within 30 days of onset of symptoms) was associated with lower hazard of death, compared to those diagnosed after 30 days (Unadjusted: HR = 0.67; 95% CI = 0.48–0.93; Adjusted: HR = 0.66; 95% CI = 0.47–0.92). There were no significant differences in terms of the drugs used for VL treatment. Irrespective of the drug, patients who completed treatment course had 97% lower risk of death (Unadjusted: HR = 0.03; 95% CI = 0.02, 0.04; Adjusted: HR = 0.03; 95% CI = 0.02, 0.05), compared to those who did not complete treatment. Tests for proportional hazards assumption (Schoenfeld residuals test), revealed that none of the CPH models had a significant p-value for the test of non-zero slope. Thus, the proportional hazards assumption could not be rejected for any of the CPH models.

**Table 2:** Hazard ratios (and 95% confidence intervals) of death among VL cases, since the time of diagnosis, from crude and adjusted Cox proportional hazards models. Bihar, 2012–13. (N = 4925).*

Characteristics	Category(s)	Hazard ratios associated with death (95% CI)	
		Unadjusted	Adjusted**
Place of treatment (Reference = Treated partly or completely at private facilities)	Treated only at public facilities	**0.52 (0.37–0.72)**	**0.61 (0.43–0.86)**
Place of VL testing (Ref = Public facility)	Private	0.98 (0.71–1.37)	0.92 (0.65–1.31)
Interval between start of VL symptoms and diagnosis (Ref = >30 days)	≤30 days	**0.67 (0.48–0.93)**	**0.66 (0.47–0.92)**
Drug received (Ref = Miltefosine)	Amphotericin-B	1.39 (0.81–2.38)	1.43 (0.82–2.51)
	Sodium stibogluconate	1.15 (0.62–2.13)	1.10 (0.57–2.10)
Completion of treatment course (Ref = No)	Yes	**0.03 (0.02–0.04)**	**0.03 (0.02–0.05)**

*Observations with missing values excluded as applicable

**All models were adjusted for age, gender, caste and principal component for socio-economic status

Numbers in bold indicate statistically significant association (*P*<0.05)

## Discussion

In the current article, we present the findings of the VL situation assessment initiative aimed at estimating the burden and mortalities associated with symptomatic VL in the Indian state of Bihar. Previous estimates of VL incidence in Bihar, which has the highest burden of VL in India, were based either on the data from public facilities or surveys conducted in limited geographical areas with high endemic foci [11, 12]. Moreover, there existed very few studies on VL-associated mortalities in India. From the perspective of India’s kala-azar elimination program, which was principally focused on prevention, understanding the correlates of VL-associated deaths could help in designing interventions targeted at reduction of VL mortalities and prioritizing deliveries of essential health services to VL patients.

The case fatality of symptomatic VL was found to be around 3%, lower than the value reported by a large scale VL mortality study conducted in Bangladesh [13]. The comparatively higher proportion of death reported in the Bangladesh study could be attributed to the large tribal ethnic population included in that study, with about 83% VL deaths occurring among tribals [13]. Moreover, unlike Bihar, about one-third of the death cases in Bangladesh did not receive any treatment. Thus, the lower mortality seen in Bihar, compared to Bangladesh, might be a consequence of wider access to modern treatment for VL.

The proportion of males were higher among VL cases. This is similar to the findings from previous studies in the same geographical region and could be attributed to their sleeping habits (sleeping outside the house) and occupational exposures (e.g. farming) [6]. Also, families of about 61% symptomatic VL patients were owners of cattle. This was not surprising given that cattle-sheds are known breeding site for the vector in Bihar [17]. For majority of patients, the interval between the onset of symptoms and diagnosis was more than 30 days. An earlier study conducted in Bihar and neighboring Nepal also reported on the issue of diagnostic delay in Bihar [18]. However, unlike the said study, most of the VL patients included in the current research sought treatment from Government-run facilities. Minimizing the delay in diagnosis and treatment, besides being prognostically beneficial for individual patients, is crucial for restricting the disease transmission as the infected hosts serve as source of leishmania amastigotes for sandflies. About 8% patients were prescribed SSG, despite the widely reported resistance against it [19]. Moreover, the likelihood of non-completion of treatment was much higher among those prescribed SSG, possibly due to adverse effects or prolonged parenteral therapy or both [20].

Probability of death was higher among people from marginalized castes corroborating the observation in Bangladesh [13]. As marginalized people in India belong mostly to lower socio-economic strata, the observed higher mortality could be an outcome of their low awareness levels and/or poorer access to healthcare [17, 21]. The impact of economic status on mortality was also evident from the fact that ownership of cattle, which is a surrogate for better economic status in rural Bihar, was found to be associated with lower mortality risk. This is not surprising as economic status is a principal determinant of access to healthcare and, in turn, disease outcome. Moreover, ownership of cattle might have been associated with milk consumption and better nutritional status, which could have potentially lowered the risk of mortality [22, 23]. Further, treatment at public facilities was found to be associated with lower risk of death compared to getting treated by private healthcare providers. In rural Bihar’s scenario, unqualified practitioners are often the mainstay of health services. Despite the fact that health services provided at public health facilities are free and there are concerns about the quality of care provided by the rural medical practitioners, residents of hard-to-reach areas often have to rely on such practitioners. Poor access to quality healthcare among VL patients in India and other resource-limited settings have long been recognized as a major concern [24]. Therefore, future policies aimed at reducing VL mortalities in India should address issues related to proper healthcare access among residents of rural areas, especially those belonging to the marginalized communities.

Proportional hazards analysis revealed that diagnosis within 30 days of symptom onset and completion of treatment course to be significant predictors of survival among symptomatic VL cases. Earlier diagnosis is likely to be associated with earlier treatment as well–which is a widely recognized determinant of survival[25]. Treatment completion, irrespective of the prescribed drug, with 96%-97% reduced risk of mortality, emerged as a strong predictor of survival. Treatment adherence, besides being essential for cure, is also important for preventing emergence of drug resistance. Therefore, targeted interventions aimed at strengthening the adherence improvement measures, such as rigorous counselling and treatment follow-up—especially for potential defaulters, need to be emphasized upon.

There were some important limitations of the present undertaking. First, the current study included only the symptomatic VL cases. However, the burden of asymptomatic VL has been reported to be much larger compared to the symptomatic cases [26]. Nevertheless, as mortality due to VL is expected to occur mostly among symptomatic cases, the mortality estimate (and its predictors) is presumed to be somewhat accurate. Second, some of the dates used in the analysis (especially date of onset of illness), an essential component of survival analysis, were approximate and based on recall. Therefore, the possibility of some amount of information bias in the estimates should be borne in mind. Third, because of unavailability of past treatment documents, it was not possible for us to determine whether a VL case was new or relapse and if there were any associated comorbidities (e.g. HIV). Another potential limitation of our findings is that our analyses could not account for all possible factors that might have contributed to mortality among VL patients (nutritional status, comorbidities etc.), as the required information were not collected. Finally, it was not possible to ascertain the exact causes of death for all cases (where no medical documents were available). However, such missing information were likely to be non-differentially distributed across the categories of the variables incorporated in the statistical model(s). Because of the aforementioned limitations, caution should be exercised while interpreting the results of our analyses (and extrapolating the findings to the national level).

Notwithstanding the limitations, the findings of this first-ever attempt at identifying the epidemiologic and health-system related correlates of VL-associated mortalities in Bihar probably could generate important insights. It was revealed that the interventions aimed at reducing mortalities should specifically target the marginalized communities and focus on improving access to early diagnostic and quality treatment services along with strengthening the treatment adherence measures. We expect our findings to contribute towards the efforts of reducing VL mortalities and help in epidemiologic transition of VL to a non-fatal low-endemic disease in Bihar.

## Supporting Information

S1 Table Source of information regarding type of drug used for VL treatment. Bihar, 2012–13 (N = 4925).
(DOCX)

S2 Table Source of identified VL cases and frequencies of cases at different stages of data compilation. Bihar, 2012–13.
(DOCX)

S3 Table STROBE Checklist (DOCX)
