# Supplementary material for: Epidemiologic Correlates of Mortality among Symptomatic Visceral Leishmaniasis Cases: Findings from Situation Assessment in High Endemic Foci in India
Source: PLoS Negl Trop Dis. 2016 Nov 21;10(11):e0005150. doi: 10.1371/journal.pntd.0005150 (PMC5117587; doi:10.1371/journal.pntd.0005150)
Supplement: S1 Table — Bihar, 2012–13 (N = 4925). (DOCX) [file pntd.0005150.s001.docx]

**S1 table. Source of information regarding type of drug used for VL treatment. Bihar, 2012-13 (N=4925).**

| **Method of determination of drug type** | **No. of cases** |
| --- | --- |
| Record-based | 2662 |
| Recollection-based | 2060 |
| No drug information available | 203 |
