# Supplementary material for: Epidemiologic Correlates of Mortality among Symptomatic Visceral Leishmaniasis Cases: Findings from Situation Assessment in High Endemic Foci in India
Source: PLoS Negl Trop Dis. 2016 Nov 21;10(11):e0005150. doi: 10.1371/journal.pntd.0005150 (PMC5117587; doi:10.1371/journal.pntd.0005150)
Supplement: S2 Table — Bihar, 2012–13. (DOCX) [file pntd.0005150.s002.docx]

**S2 table. Source of identified VL cases and frequencies of cases at different stages of data compilation. Bihar, 2012-13.**

| **Stage of data collection** | | **No. of cases** |
| --- | --- | --- |
| **(A)** Total listed cases in the public facilities (diagnosed between January 2012 and June 2013) | | 5770 |
| **(B)** Public facility listed cases successfully traced to their residence and interviewed | | 4962 |
| **(C)** Additional cases traced and interviewed **[C1 + C2]** | | 1119 |
|  | **(C1)** Snowballing | 262 |
|  | **(C2)** Scanning of private providers | 857 |
| **(D)** Total cases interviewed **[B + C]** | | 6081 |
| **(E)** Total eligible cases **[E1 + E2 + E3]** | | 5432 |
|  | **(E1)** Case definition 1* | 4493 |
|  | **(E2)** Case definition 2* | 857 |
|  | **(E3)** Case definition 3* | 82 |
| **(F)** Cases used for mortality analysis | | 4925 |

*Case definition 1 = Identified from line list of public facilities; Case definition 2 = Possession of documents confirming VL diagnosis (excluding the cases meeting case definition 1); Case definition 3 = Possession of documents related to VL treatment (excluding the cases meeting case definitions 1 and 2).
