# Supplementary material for: Epidemiologic Correlates of Mortality among Symptomatic Visceral Leishmaniasis Cases: Findings from Situation Assessment in High Endemic Foci in India
Source: PLoS Negl Trop Dis. 2016 Nov 21;10(11):e0005150. doi: 10.1371/journal.pntd.0005150 (PMC5117587; doi:10.1371/journal.pntd.0005150)
Supplement: S3 Table — (DOCX) [file pntd.0005150.s003.docx]

Checklist S1: STROBE Checklist

|  | Item No | Recommendation | Y/N |
| --- | --- | --- | --- |
| Title and abstract | 1 | (a) Indicate the study’s design with a commonly used term in the title or the abstract | N |
|  |  | (b) Provide in the abstract an informative and balanced summary of what was done and what was found | Y |
| Introduction |  |  |  |
| Background/rationale | 2 | Explain the scientific background and rationale for the investigation being reported | Y |
| Objectives | 3 | State specific objectives, including any prespecified hypotheses | Y |
| Methods |  |  |  |
| Study design | 4 | Present key elements of study design early in the paper | Y |
| Setting | 5 | Describe the setting, locations, and relevant dates, including periods of recruitment, exposure, follow-up, and data collection | Y |
| Participants | 6 | (a) Cross-sectional study—Give the eligibility criteria, and the sources and methods of selection of participants | Y |
| Variables | 7 | Clearly define all outcomes, exposures, predictors, potential confounders, and effect modifiers. Give diagnostic criteria, if applicable | Y |
| Data sources/ measurement | 8* | For each variable of interest, give sources of data and details of methods of assessment (measurement). Describe comparability of assessment methods if there is more than one group | Y |
| Bias | 9 | Describe any efforts to address potential sources of bias | Y |
| Study size | 10 | Explain how the study size was arrived at | N |
| Quantitative variables | 11 | Explain how quantitative variables were handled in the analyses. If applicable, describe which groupings were chosen and why | Y |
| Statistical methods | 12 | (a) Describe all statistical methods, including those used to control for confounding | Y |
|  |  | (b) Describe any methods used to examine subgroups and interactions | N |
|  |  | (c) Explain how missing data were addressed | N |
|  |  | (d) Cross-sectional study—If applicable, describe analytical methods taking account of sampling strategy |  |
|  |  | (e) Describe any sensitivity analyses | N |
| Participants | 13* | (a) Report numbers of individuals at each stage of study—eg numbers potentially eligible, examined for eligibility, confirmed eligible, included in the study, completing follow-up, and analysed | Y |
|  |  | (b) Give reasons for non-participation at each stage | N |
|  |  | (c) Consider use of a flow diagram | N |
| Descriptive data | 14* | (a) Give characteristics of study participants (eg demographic, clinical, social) and information on exposures and potential confounders | Y |
|  |  | (b) Indicate number of participants with missing data for each variable of interest | N |
| Outcome data | 15* | Cross-sectional study—Report numbers of outcome events or summary measures | Y |
| Main results | 16 | (a) Give unadjusted estimates and, if applicable, confounder-adjusted estimates and their precision (eg, 95% confidence interval). Make clear which confounders were adjusted for and why they were included | Y |
|  |  | (b) Report category boundaries when continuous variables were categorized | Y |
|  |  | (c) If relevant, consider translating estimates of relative risk into absolute risk for a meaningful time period | N |
| Other analyses | 17 | Report other analyses done—eg analyses of subgroups and interactions, and sensitivity analyses | N |
| Discussion |  |  |  |
| Key results | 18 | Summarise key results with reference to study objectives | Y |
| Limitations | 19 | Discuss limitations of the study, taking into account sources of potential bias or imprecision. Discuss both direction and magnitude of any potential bias | Y |
| Interpretation | 20 | Give a cautious overall interpretation of results considering objectives, limitations, multiplicity of analyses, results from similar studies, and other relevant evidence | Y |
| Generalisability | 21 | Discuss the generalisability (external validity) of the study results | Y |
| Other information |  |  |  |
| Funding | 22 | Give the source of funding and the role of the funders for the present study and, if applicable, for the original study on which the present article is based | Y |
